# Aortic Dissection as the Culprit for Seizure

**DOI:** 10.7759/cureus.17325

**Published:** 2021-08-20

**Authors:** Fernando Rivera-Alvarez, Marvi Qureshi, Anines Quinones, Ayanna Walker, Latha Ganti

**Affiliations:** 1 Emergency Medicine, University of Central Florida, Orlando, USA; 2 Emergency Medicine, Osceola Regional Medical Center, Kissimmee, USA; 3 Emergency Medicine, HCA Healthcare Graduate Medical Education Consortium Emergency Medicine Residency Program of Greater Orlando, Orlando, USA; 4 Emergency Medicine, Envision Physician Services, Plantation, USA; 5 Emergency Medicine, University of Central Florida College of Medicine, Orlando, USA

**Keywords:** aortic dissection, syncope, stanford type a dissection, debakey classification, emergency medicine

## Abstract

The authors present a case of syncope that was caused by aortic dissection. As the main artery to the system circulation, aortic dissection can manifest in numerous ways. This case reminds the clinician of the high index of suspicion necessary to diagnose this potentially life-threatening problem. Indeed, the mortality rate increases 2% per hour in the first 24 hours for a type A aortic dissection, so prompt diagnosis is imperative. The diagnostic complexity in this case is compounded by the very broad differential diagnosis for both aortic dissection and syncope.

## Introduction

Aortic dissection affects 5-30 patients per 1,000,000 every year with a high mortality rate [1]. It occurs when there is a tear in the intima of the aorta, which causes the blood flowing throughout the body to split the layers of the aorta apart [2]. This leads to a “false lumen” that is created between the aortic intima and the media or adventitia [3]. This can further progress both proximally and distally from the initial point of tear, which can further lead to tissue ischemia, intravascular volume loss, cardiac tamponade, myocardial infarction, and aortic regurgitation [2].

There are two different classifications of aortic dissection, the most common one being the Stanford classification [2]. Stanford type A involves any dissection of the ascending aorta, and type B involves a dissection that does not include the ascending aorta [2]. In terms of management, type A dissections are surgical emergencies and type B aortic dissections are medically managed [2]. The mortality rate increases 2% per hour for a type A dissection [4].

Risk factors for aortic dissection include a connective tissue disease, family history of aortic disease, and a known thoracic aortic aneurysm, none of which the patient had [4]. Patients can present with a wide variety of symptoms, making the diagnosis for aortic dissection difficult [5]. Patients usually complain of acute chest or abdominal pain, with some experiencing neurological symptoms upon initial presentation [6]. This is more common for patients who have a dissection that extends into the carotid arteries, in which they may present with stroke-like symptoms, altered mental status, or syncope [2].

The neurological complications of aortic dissection include neurological deficits similar in presentation to an ischemic stroke or coma, transient ischemic attack, spinal cord ischemia (involving paraplegia), ischemic neuropathy, as well as hypoxic-ischemic encephalopathy [4]. Other complications include syncope, generalized tonic-clonic seizures, somnolence, and altered mental status [5]. In this article, the authors discuss a patient who presented with altered mental status due to aortic dissection.

## Case presentation

A 49-year-old male with a medical history of hypertension, dyslipidemia, and prediabetes was brought to the emergency department (ED) by Emergency Medical Services (EMS) who received a call for an unconscious patient. The patient’s wife stated that he woke up that morning in his normal state of health and went to take a shower. Moments later, his wife heard a noise coming from the bathroom. She had difficulty opening the door because the patient’s body was against it. She found him unresponsive, with stiff arms, legs extended, jaw clenched, and foam in his mouth. His wife then started cardiopulmonary resuscitation as the son alerted EMS.

On arrival, EMS found the patient in the hallway outside of the bathroom. EMS inquired if the patient had a seizure disorder or history of drug use, and per the family, he did not. The patient was unable to provide any information due to his condition. At the time of initial contact, the patient had a strong carotid pulse, his teeth were clenched, and he was tachypneic with equal chest rise and fall. His lungs were clear with bilateral ventilation, and there was no accessory muscle use noted. Other physical examination findings included warm and dry skin, rigid arms and legs, pupils equal, dilated, and nonreactive, and a Glasgow Coma Scale (GCS) of 6. Upon further head to toe assessment, there were no obvious injuries, trauma, or unusual findings. There was a prominent waveform on capnography, the patient’s blood glucose level was 111 g/dL, and a 12-lead electrocardiogram (ECG) showed sinus bradycardia without acute ST segment elevation or depression. At this moment, seizure was the most likely diagnosis.

As the EMS crew evaluated the patient in the traditional airway, breathing, circulation (ABC) sequence, there were many difficulties encountered. EMS was unable to initially assess his airway due to clenched teeth. They were unable to obtain an automated blood pressure or true oxygen saturation due to excessive movement and rigidity in his extremities. They also had difficulty obtaining intravenous access because of his rigid posture. There were several attempts made to address the ABC sequences despite these shortcomings. The patient was given supplemental oxygen via nasal cannula, and later, the airway was manually opened and suctioned due to gurgling respirations. Just a small amount of saliva was cleared and the gurgled respirations resolved. A nasopharyngeal airway device was inserted to facilitate an open airway with no change in the patient’s status. Although a strong radial pulse was noted, an official blood pressure was not able to be obtained. Finally, in an effort to address his rigidity, midazolam was given intranasally but did not result in improvement.

Upon arrival to the emergency department, the patient presented with altered mental status, labored breathing, and intermittent decerebrate posturing. His GCS had declined to 4. The patient was intubated using a video laryngoscopy with direct visualization of the vocal cords. Endotracheal tube placement was confirmed by adequate color change on colorimetric device and bilateral breath sounds on auscultation.

The ECG was taken without any acute ischemic changes, no wide QRS, no arrhythmias, no prolonged QT, normal intervals, and no acute ST elevations. There were, however, diffuse, nonspecific, symmetric T wave inversions. A computed tomography (CT) scan of the head and brain without contrast was performed and was negative for any intracranial process. Laboratories studies revealed no acute abnormal values requiring immediate intervention. There were no signs of infection or electrolyte abnormalities, and the troponin was negative. The patient’s systolic blood pressure was 115 mmHg after 2 liters of normal saline, and throughout this time, he had a normal heart rate.

Due to onset of the events, altered mental status and rigidity, a stroke alert was called for immediate neurological evaluation. The patient did not show any focal neurological signs, and his National Institutes of Health Stroke Scale (NIHSS) was 38. He was taken back to CT to complete the stroke assessment which included a CT angiogram (CTA) head and neck and a CT brain perfusion. While there, the patient became mildly hypotensive, dropping his mean arterial pressure to 60 mmHg with an oxygen saturation of 92%. At this point, the decision to add a CTA of the chest was made to rule out a pulmonary embolism.

**Figure 1:**
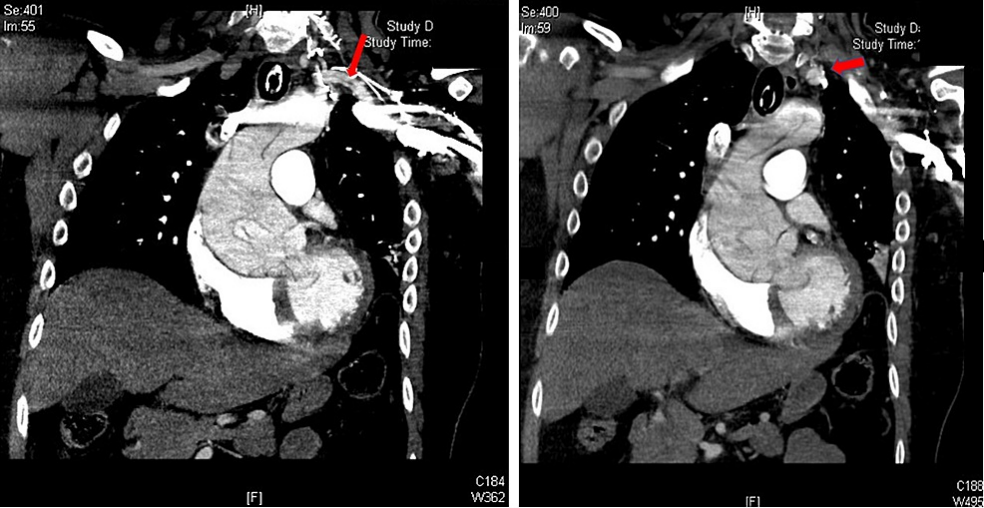
CT angiogram demonstrating Stanford type A aortic dissection (arrows) H: head, F: foot.

CTA chest did not reveal any main or segmental pulmonary emboli but rather revealed a Stanford type A aortic dissection extending into the left proximal subclavian artery and a 6 cm aortic root aneurysm. The dissection extended into the great vessels including brachiocephalic, left common carotid and left subclavian arteries (Figure 1).

**Figure 2:**
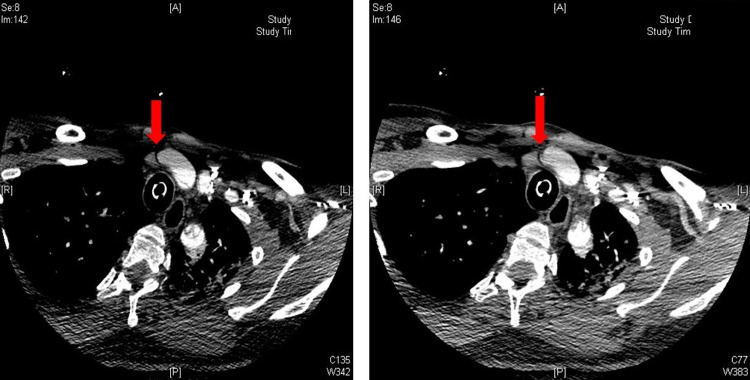
CT angiogram of the neck showing occlusion at the origin of the right common carotid artery (arrows) A: anterior, P: posterior.

CTA of brain showed sulci that were less conspicuous when compared with the baseline CT examination and loss of gray-white matter differentiation suggesting generalized edema and evolving mass effect. CTA of the neck showed that occlusion at the origin of the right common carotid artery was noted, with dissection extending into the left common carotid artery with subtotal occlusion and thrombus formation (Figure 2).

**Figure 3:**
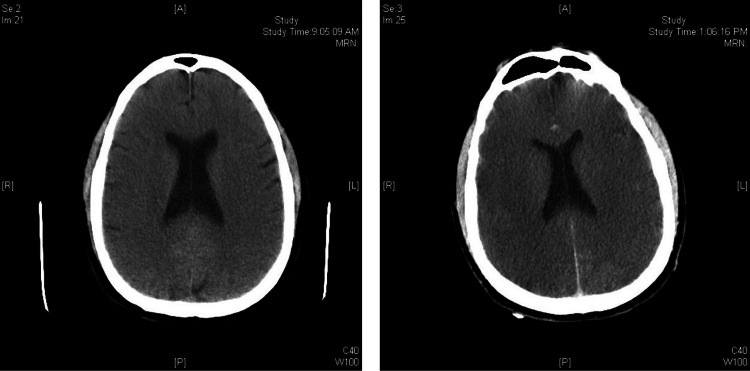
Brain CT showing interval worsening and bilateral cerebral edema, worrisome for hypoxic injury A: anterior, P: posterior, R: right, L: left.

The case was discussed in detail with the cardiothoracic surgeon who determined that since the patient had a poor neurologic examination, he was not a surgical candidate at the time. The case was also discussed in detail with the neurology and the cardiovascular ICU (CVICU) teams. Attendings of both services were at bedside evaluating the patient and speaking with the family. The patient was admitted to the CVICU. The brain CT was repeated five hours after arrival which showed interval worsening in diffuse bilateral cerebral edema worrisome for hypoxic injury (Figure 3).

 The patient’s family opted for comfort care given the patient’s poor prognosis.

## Discussion

The authors review the case of a middle-aged man with an aortic dissection who presented with alerted mental status and decerebrate posturing. The diagnosis was difficult because the patient did not present with chest pain and, in fact, only presented with neurological symptoms.

Aortic dissection involves aortic blood entering the middle layer of the aortic wall through intimal rupture [4]. This can then extend and dissect along the longitudinal axis of the aorta [4]. A major risk factor is hypertension. Other risk factors include atherosclerosis, trauma, congenital aortic valve abnormalities, and Marfan syndrome [7]. Patients present with symptoms depending on the location of the affected blood vessels as well as the anatomical structures nearby [4].

There are two types of classification of aortic dissection, the Stanford and the Debakey. The Stanford classifies ascending aortic dissections as type A and descending aortic dissection as type B. Surgery is the treatment for type A dissection; however, it carries a 31%-46% risk of morbidity or mortality in patients with cerebral infarction [8]. One factor that is more likely to indicate a poor outcome following surgery is a poor neurological status, as seen in our patient [8].

Studies show that temporary and persistent neurological symptoms are present in 17% to 40% of aortic dissection patients, which may lead to misdiagnosis, especially if they do not have chest pain as their primary presenting symptom [4]. Half of the patients who did not report pain upon initial presentation only had neurological findings [9]. Imaging is the mainstay of diagnosing aortic dissection. This involves thoracic and abdominal CT scans as well as CT angiography [4].

## Conclusions

Aortic dissection should be a consideration in patients who present with syncope, seizure, or altered mental status. This is important from an emergency standpoint since, depending on the type of dissection and specifications, the patient may need to be taken to the operating room immediately. Any time delay may impact the patient’s chance of surviving. In addition, if a stroke is considered as the cause for syncope or altered mental status and tissue plasminogen activator (tPA) is given, this would be detrimental if there were an underlying aortic dissection. As such, it is important to incorporate CT chest as part of initial CT imaging sequence and protocols of unexplained acute neurological presentations to expedite the diagnosis of aortic dissection. 
